# Bioactivity Profiling of Chemical Mixtures for Hazard Characterization

**DOI:** 10.1021/acs.est.4c11095

**Published:** 2024-12-20

**Authors:** Xiaojing Li, Jiarui Zhou, Yaohui Bai, Meng Qiao, Wei Xiong, Tobias Schulze, Martin Krauss, Timothy D. Williams, Ben Brown, Luisa Orsini, Liang-Hong Guo, John K. Colbourne

**Affiliations:** † Centre for Environmental Research and Justice (CERJ), School of Biosciences, The University of Birmingham, Birmingham B15 2TT, U.K.; ‡ Research Centre for Eco-Environmental Sciences, Chinese Academy of Sciences, Beijing 100085, P. R. China; § Key Laboratory of Environmental Biotechnology, Research Centre for Eco-Environmental Sciences, Chinese Academy of Sciences, Beijing 100085, P. R. China; ∥ Department Exposure Science, Helmholtz Centre for Environmental Research − UFZ, 04318 Leipzig, Germany; ⊥ Environmental Genomics and Systems Biology Division, Lawrence Berkeley National Laboratory, Berkeley 94720, United States; # The Alan Turing Institute, British Library, London NW1 2DB, U.K.; ∇ Hangzhou Institute for Advanced Study, UCAS, Hangzhou, Zhejiang 310020, P. R. China

**Keywords:** freshwater, chemical mixtures, transcriptomics, biomolecular effect data, new approach methodologies (NAMs)

## Abstract

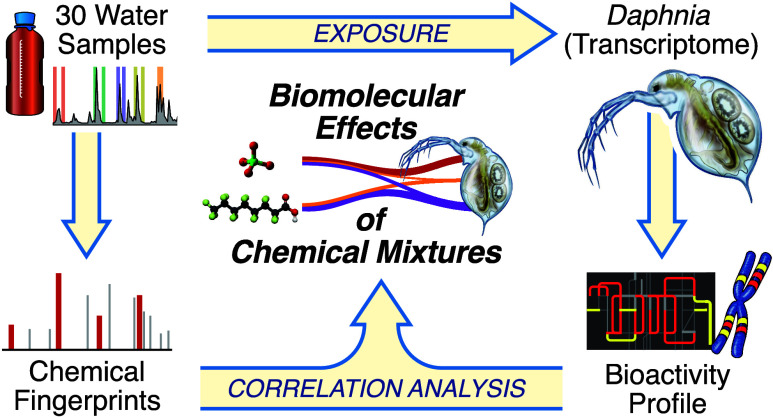

The assessment and regulation of chemical toxicity to protect human health and the environment are done one chemical at a time and seldom at environmentally relevant concentrations. However, chemicals are found in the environment as mixtures, and their toxicity is largely unknown. Understanding the hazard posed by chemicals within the mixture is critical to enforce protective measures. Here, we demonstrate the application of bioactivity profiling of environmental water samples using the sentinel and ecotoxicology model species *Daphnia* to reveal the biomolecular response induced by exposure to real-world mixtures. We exposed a *Daphnia* strain to 30 sampled waters of the Chaobai River and measured the gene expression response profiles. Using a multiblock correlation analysis, we establish correlations between chemical mixtures identified in 30 water samples with gene expression patterns induced by these chemical mixtures. We identified 80 metabolic pathways putatively activated by mixtures of inorganic ions, heavy metals, polycyclic aromatic hydrocarbons, industrial chemicals, and a set of biocides, pesticides, and pharmacologically active substances. Our data-driven approach discovered both known bioactivity signatures with previously described modes of action and new pathways linked to undiscovered potential hazards. This study demonstrates the feasibility of reducing the complexity of real-world mixture toxicity to characterize the biomolecular effects of a defined number of chemical components based on gene expression monitoring of the sentinel species *Daphnia*.

## Abstract 2

Understanding how chemical mixtures affect organisms is essential for protecting environmental health. We present a data-driven, hypothesis-free approach that characterizes hazards of complex chemical mixtures in the environment.

## Introduction

Chemical pollution is a global threat to public health^1^ due to unregulated mixtures from domestic, agricultural and industrial processes entering the environment. Freshwater ecosystems, especially rivers, are particularly impacted, as they receive untreated and treated wastewater from domestic and industrial effluents.^2^ This is because conventional wastewater treatment processes are not designed to effectively remove industrial, agricultural and domestic pollutants from wastewater.^3^ These chemicals enter the food chain and water supply through irrigation and aquifer recharges, adversely affecting environmental and human health.^4^

Current regulatory frameworks assess chemical safety one substance at a time, neglecting the cumulative toxicity of chemical mixtures.^5,6^ The total number of chemicals tested by 2022 was 12,714,^7^ corresponding to less than 0.2% of the chemicals potentially present in the environment. The low percentage of tested chemicals can be explained by the low throughput of traditional risk assessments, which require animal testing.

New approach methodologies (NAMs) in regulatory toxicology include bioactivity measurements (e.g., genes and metabolites) of changes induced by exposure to chemicals. They are regarded as high-throughput alternatives to traditional methods, are suitable for detecting sublethal effects of chemical and chemical mixtures^8,9^ and enable the grouping of chemicals based on their bioactivity.^10^ This helps define points of departure to adversity^11^ and classify substances by their modes of action (MoAs).^12^ However, despite their advantages, regulatory frameworks have been reluctant to adopt NAMs due to perceived scientific, technical, regulatory, and economic challenges.^13^ Validating the robustness and advantages of NAMs in real-world cases can help build confidence in their use and create harmonized guidelines for their application.

We recently published a conceptual framework that uses gene expression monitoring to identify correlations between ambient chemical mixtures and biomolecular responses in the sentinel species *Daphnia*.^14^ This framework enables *Daphnia* to be used as an early warning system of the chemical hazards by profiling gene expression responses to chemical mixtures and identifying hazardous chemicals within mixtures. Here, we demonstrate the use of this framework in a case study by exposing *Daphnia magna* to surface river waters from the Chaobai River system in China. These water samples are chemically complex as the river system receives chemical pollutants from agricultural runoffs, domestic reclaimed waters, and industrial wastewater effluents.^15,16^ We characterized the chemical fingerprints of 30 samples collected along the river, using target and target screening methods. We exposed a commercial strain of*D. magna*to these water samples, conducting both immobilization assays and genome-wide transcriptome profiling. We combined gene coexpression network analysis with multiblock correlation analysis to link chemicals within the environmental mixtures and the bioactivity profiles in*D. magna*, revealing networks of genes dysregulated by chemicals. By applying pathway overrepresentation analysis, we identified enriched functional pathways associated with those chemicals. We benchmarked our findings by identifying known associations between metabolic pathways and chemicals from published studies and discovered new toxicity pathways. The identification of known pathways through this data-driven approach provides confidence in the newly discovered associations between chemicals and novel coresponsive pathways. This data-driven approach for integrating analytical chemical analysis and bioactivity profiling enhances our ability to unravel the hazards of environmental chemical mixtures, paving the way to more informed and effective environmental risk assessment.

## Materials and Methods

### Field Sampling Site and Strategy

The Chaobai River system, the second longest river in the Beijing area, spans 458 km and covers a drainage area of 13,846 km^2^^17^ across the Hebei-Beijing-Tianjin region. It is formed by three rivers, namely the Bai, Chao and Chaobai Rivers. The Bai River and the Chao River both originate from Yunwu Mountain in Hebei province. They converge to form the Chaobai River, which flows through populated towns in northern Beijing and agricultural lands in Tianjin before reaching the Pacific Ocean. This river system is impacted by industrial effluents from various sectors, including food, cosmetics, pharmaceuticals, and automotive manufacturing.^18^ The downstream (Chaobai River) receives reclaimed water from the Wenyu River, contributing up to 38 billion cubic meters to its annual flow.^19^ Additionally, wastewater treatment plants along this river release 9.2 billion cubic meters of treated effluents into the water flow yearly.^20^

**Figure 1:**
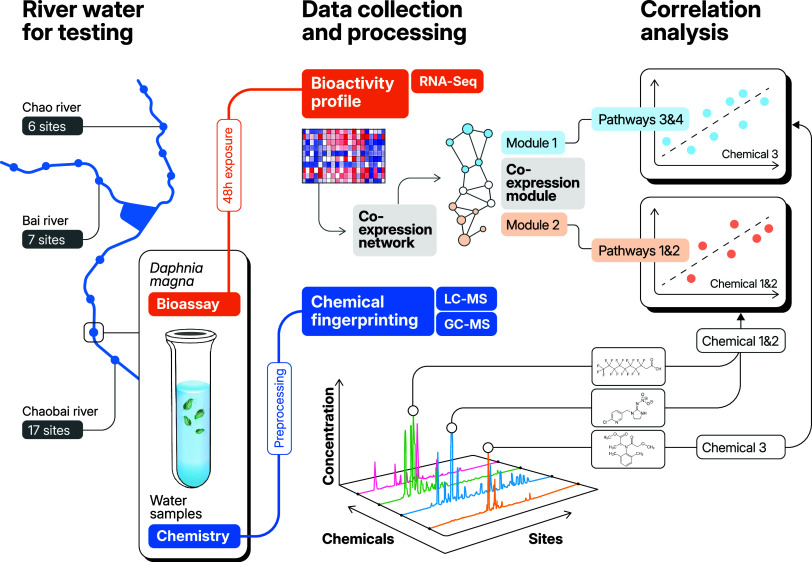
Workflow of the Chaobai case study. A total of 30 water samples were collected from three rivers: the Chao River (6 sites), the Bai River (7 sites), and the Chaobai River (13 sites). These samples were used for chemical fingerprinting and exposure assays with a commercial strain of *Daphnia magna*. Exposed *Daphnia* that were not immobilized after exposure were used for transcriptome profiling (RNA-Seq). Genome-wide gene expression was used to generate coexpression gene modules. Multiblock correlation analysis was then used to identify correlations between chemicals within mixtures and pathways enriched within coexpression modules.

For this study, we collected water from seven locations along the Bai River, six locations along the Chao River, and 17 from the Chaobai River (Figure 1). The pH and total dissolved solids were measured *in situ* using a multiparameter water quality probe (MYRON Co.) (Table S1). From each location, ∼ 8L of surface water was collected in Duran amber glass bottles and transported to the laboratory at room temperature (19–23 °C). Of the total collected water per sample, 500 mL was used for immobilization assays with *D. magna*, and the remaining volume was used for chemical fingerprinting.

### Chemical Fingerprinting of the Chaobai Water Samples

The chemical fingerprints of the Chaobai water samples were generated by target and target screening analyses (Table S2). The target analysis measured inorganic elements and polycyclic aromatic hydrocarbons (PAHs), while the target screening analysis quantified polar organic compounds. Altogether, we measured 609 chemicals, of which 385 were detected.

#### Target Chemical Analysis

The target chemical analysis was used to quantify 19 inorganics (e.g., P, N and heavy metals) and 16 PAHs (Table S2). For this analysis, water samples were filtered through 0.45 μm glass fiber membrane (GF/F, Whatman) and stored at 4 °C before analysis. Total nitrogen (TN), ammonia (NH_4_^+^), nitrate (NO_3_^–^) and nitrite (NO_2_^–^) were measured with UV spectrophotometry after alkaline potassium persulfate digestion, while total phosphorus (TP) and phosphate (PO_4_^3–^) were quantified using ammonium molybdate spectrophotometry, following methods described in Liao et al.^21^ Heavy metal concentrations (Cd, Cr, Cu, Ni, Zn, Pb, and As) were determined using inductively coupled plasma-mass spectrometry (ICP-MS, 7500a, Plasma Quad 3), while concentrations of K, Ca, Na, and Mg were assessed via inductively coupled plasma optical emission spectrometry (ICP-OES, OPTIMA 2000, PerkinElmer), following methods described in Xiong et al.^17^ Sixteen PAHs were quantified using an Agilent 7890A gas chromatographer coupled with a 5795C mass spectrometry detector with electrospray ionization sources in the selective ion monitoring mode following methods described in Qiao et al.,^19^ with detailed methods outlined in Supporting Information Section A.

#### Target Screening Analysis of Polar Organic Substances

The target screening analysis quantified 574 organic chemicals (Table S2), including pharmaceuticals, pesticides, biocides, industrial solvents, *etc*. For this analysis, two liters of surface water were filtered through a 0.7 μm glass microfiber membrane (GF/F, Whatman) and extracted using solid-phase extraction with HLB cartridges (500 mg, 6 mL, Waters), which were preconditioned with methanol and deionized water. Each sample underwent positive and negative ion modes analysis by liquid chromatography-high resolution mass spectrometry (LC-HRMS), as also described in Section A. The conversion of raw mass spectral data (mzML) and centroiding was accomplished using ProteoWizard, incorporating the instrument’s built-in library.^22^ Data processing, including peak picking, alignment, gap filling, and peak annotation, was performed using MZmine 2.52 (http://mzmine.github.io). The annotated peak list underwent further analysis with an in-house R-package, MZquant (version 0.7.22), for semiautomated quantification. A final cleanup of the annotated peaks, blank filtering, automatic internal standard assignment, and peak quantification was completed using MZquant (version 0.7.22). Compounds with broad peaks or high background noise, which could not be quantified through the semiautomatic workflow, were manually annotated with TraceFinder 4.1 (Thermo Scientific). The raw peak tables were refined based on final peak annotations following assessments by MZquant and TraceFinder.

#### Data Preprocessing and Analysis

Chemicals detected in target and target screening assays that were below detection limits or absent in more than 50% of the samples were excluded from downstream analysis. This was done to meet the minimum requirements for missing value imputation. The *k*-nearest neighbor algorithm (*k* = 5)^23^ was used for imputing missing values of 215 chemicals retained after the filtering process. The principal component analysis (PCA) plot was used to reveal the similarity of chemical fingerprints based on the standardized and normalized concentration values of 215 selected chemicals.

### Bioactivity Profiling of Daphnia Exposed to the Chaobai Water Samples

We performed immobilization assays and genome-wide transcriptome profiling to quantify the *Daphnia magna’s* biological and biomolecular responses to chemical mixtures from the Chaobai water samples.

#### Immobilization Assays

Surface waters collected from 30 sites were filtered using the 0.45 μm glass fiber membrane (GF/F, Whatman) to remove suspended particles and bacteria. The filtered water samples were then frozen at −20 °C prior to exposure to reduce any bacterial or viral bioactivities. The filtered water samples were acclimated at 20 °C overnight prior to OECD immobilization assays, where immobilization is recorded as an apical end point.^24^ Three biological replicates of the *D. magna* commercial strain IRCHA 5 (Water Research Centre, Medmenham, U.K.), each containing five 24 h-old daphnids, were used per sample. *Daphnia* were exposed to river waters filtered by a 0.7 μm glass fiber membrane (GF/F, Whatman). After 48 h of exposure, the number of immobilized daphnids was counted, whereas the mobile daphnids were collected, flash-frozen in liquid nitrogen and stored at -80 °C until transcriptome profiling.

#### Transcriptome Profiling

Flash-frozen daphnids from each exposure were homogenized for total RNA extraction using the Agencourt RNAdvance Tissue Total RNA kit (Beckman Coulter), following the manufacturer’s instructions. The concentration and the quality (integrity and purity) of the extracted RNA were quantified with the Nanodrop 8000 Spectrophotometer (Labtech Ltd., U.K.) and the TapeStation 2200 (Agilent Technologies), respectively. The mRNA was prepared into cDNA libraries using the NEBNext Ultra Directional RNA Library Prep Kit (New England Biolab E7420L) with NEBnext Multiplex Oligos for Illumina Dual Index Primers (New England Biolabs E7600S) RNA extraction and library preparation were performed with the Biomek FxP workstation (Beckman Coulter A31842). The quality of the constructed libraries was assessed using the Tapestation 2200. Libraries were pooled at a final molarity of 3 nM and 100-bp paired-ended sequenced on a HiSeq4000 (Illumina) at the Beijing Genomics Institute (BGI), aiming for 5 million reads per sample.

#### Sequence Preprocessing

RNA sequences were trimmed with Trimmomatic (version 0.32)^25^ to remove adapter sequences and retain reads with an average Phred score ≥30 with FastQC (version 0.11.9). High-quality reads were mapped on the *D. magna* reference genome^26^ using STAR (version 2.7.10a), and the expression levels of the mapped genes were quantified by RSEM (version 1.3.2) using default settings.^27^ Gene counts were preprocessed in R (version 4.0.3) with a customized script to exclude genes with average read counts <5, total counts <10, or zero counts in more than 60% of the samples. The count table of filtered genes was normalized using the conditional quantile normalization approach provided by the “cqn” package (version 6.0.0).^28^ The normalized gene counts were then used to perform similarity analysis with PCA, construct coexpression network, and multiblock correlation, as explained in the following.

### Linking the Chemical Fingerprint to the Bioactivity Profile

*Gene coexpression module identification*. We identified coexpression modules (clusters of genes with covarying expression patterns) with a customized computational script. We conducted 50 permutations, which included two steps in each run: first, we calculated Pearson correlations based on bootstrapped data with noise; second, we applied threshold on correlation coefficients to ensure that the coexpression network follows a scale-free topology, with a detailed explanation of diagnostic plots presented in the Supporting Information section A. After that, we ensembled results from 50 runs to construct a coexpression network. We then used the “infomap” algorithm^29^ to identify gene clusters with similar expression patterns across 30 water samples, hereafter called coexpression modules.

#### Multiblock Correlation Analysis

To identify correlations between chemical fingerprints and bioactivity profiles, we used the ‘Sparse Generalized Canonical Correlation Analysis’ (SGCCA) algorithm in the RGCCA package (version 2.1.2).^30,31^ This approach identifies correlations between variations of chemical substances within mixtures and variations of genes within modules on a low-dimensional latent space. In this latent space, projected gene and chemical vectors, so-called gene and chemical low-dimension representations (LDRs), are linearly correlated. The relative contribution of individual chemicals and genes to the corresponding LDRs is measured by squared weights (SWs); the sum of SWs of all chemicals or genes selected per LDR equals 1.

#### Module Enrichment Analysis

Using a Mann–Whitney *U* test, an enrichment analysis was conducted to identify the coexpression module consisting of genes with significantly larger SWs than genes not within the module. The resulting P-values were adjusted for a false discovery rate of 0.05 using the Benjamini-Hochberg approach. This analysis provided a list of significantly enriched coexpression modules for each LDR.

#### Pathway Overrepresentation Analysis of Significantly Enriched Coexpression Modules

We performed a pathway overrepresentation analysis on each significantly enriched coexpression module per LDR, using the KEGG Pathway database.^32^ This enabled the identification of overrepresented metabolic pathways per module and LDR. Significantly enriched pathways (adjusted *P*-value <0.05) were identified following a chi-square test adjusted for multiple testing using the Benjamini-Hochberg procedure.

## Results and Discussion

### Diversity and Distribution of Chemical Pollutants among the Sampling Sites

The chemical fingerprints of 30 river samples detected and quantified 385 chemical substances. As shown in Figure S1, a large proportion of the variance in the chemical fingerprints separated the upstream (Bai River and Chao River) from the downstream sites (Chaobai River) (31.4% of variance); conversely, 9.8% of the variance separated sites within the rivers. The upstream sites clustered closer together, indicating a higher similarity in the chemical mixtures in upstream than downstream waters past their confluence point (Figure S1). This could be explained by the ongoing impacts of domestic pollution and intensive farming activities across the downstream sites.^16^

Of the 385 chemicals detected, 215 were present at more than 15 sites, including 16 inorganic compounds (7 were heavy metals), 15 polycyclic aromatic hydrocarbons (PAHs), and 184 polar organic compounds (Table S2). Additionally, 44 chemicals were exclusive to a single sampling site, whereas 90 were ubiquitously detected across all 30 sites (Table S2). The 215 chemicals detected at more than 15 sites, could be grouped in 19 chemical classes, including PAHs, perfluorinated compounds (PFCs), pharmaceuticals and pesticides (Figure S2). Fifteen PAHs were detected across the three rivers. Four PFCs were prevalently detected in downstream river sites, including perfluorooctanoic acid (PFOA), perfluorooctanesulfonic acid (PFOS), perfluoroheptanoic acid, and 6:2 fluorotelomer sulfonic acid; these PFCs are known as “forever compounds” due to their persistence in the environment, and they primarily come from industrial discharges, landfill leachate, and atmospheric deposition.^33^ Out of 33 pharmaceuticals commonly detected across the 30 river sites, 17 were consistently detected in downstream waters (specifically in the Chaobai River). Six pharmaceuticals (i.e., n-acetyl-4-aminoantipyrine, amantadine, budesonide, dimethylamino phenazone, diazepam, and 4-formyl-antipyrine) were detected in all 30 sites, likely resulting of untreated domestic wastewater entering the rivers.^15^ It is possible that some of the detected compounds are transformation products of parent compounds. Of the 51 pesticides detected in more than 15 sites, 20 were common to all downstream sites of the Chaobai River (Table S2). These pesticides and their transformation products likely originate from agricultural runoffs from farmlands^34^ along the Chaobai River. Many of the pesticides found in our study are also found in other rivers in China due to their wide application in controlling weeds in crops.^35,36^

Quantifying 385 chemical substances allowed us to generate a comprehensive chemical fingerprinting of the water samples using target and target screening approaches. However, this number is likely an underestimation of the total number of chemical compounds present in the Chaobai River. Further refinement of detection methods and broader analytical coverage are needed to capture the full complexity of the chemical mixtures in these waters.

### Bioactivity Profiles of the Chaobai Water Samples

Although no acute toxicity (immobilization) was observed in the *Daphnia* exposed to the Chaobai water samples, we observed biomolecular responses through variations in the expression of 19,942 genes. Following preprocessing (filtering) steps described in the methods, 10,440 genes were retained for downstream analysis. With these genes, we built a coexpression network to identify gene coexpression modules. A total of 22 modules (including 7608 genes) were identified through this approach, comprising between 23 (module 22) and 1026 genes (module 1) (Figure S3).

The PCA plot based on 10,440 genes showed no clear partitioning of the data by location and/or river (Figure S4), which was unexpected given the partitioning of the chemicals by rivers (Figure S2). This suggests that the variation in the mixture composition between sites, as revealed by quantifying hundreds of chemicals, is not reflected in the changes in the expression of individual genes. This result differs from two previous studies using *Daphnia magna’s* transcriptomic profiles to assess biomolecular responses to surface water samples.^37,38^ However, those previous studies measured physicochemical parameters (e.g., pH, temperature, suspended solids) and a limited number of inorganic substances (e.g., TN, TP, As, Cr, *etc*.). It is plausible that other environmental factors not considered in our study (e.g, dissolved oxygen, salinity, humic substances) may have influenced the bioavailability of the chemicals studied. However, the absence of distinct gene-specific responses across river sites is not unexpected. Many of the quantified chemicals are present at trace levels, which are unlikely to elicit strong, gene-specific expression changes. Instead, these chemicals are more likely to induce subtle, coordinated changes in the expression of multiple genes. These changes, while biologically relevant, may fall below the statistical detection threshold, potentially explaining the lack of significant gene-by-site partitioning observed in the PCA plot (Figure S4). Therefore, we used the coexpression patterns among genes to discover covariation with ambient chemical mixtures in the Chaobai River system.

### Correlation Analysis via SGCCA

To correlate coexpression modules of bioactivity profiles with ambient chemical mixtures, we used ‘Sparse Generalized Canonical Correlation Analysis’ (SGCCA). This correlation analysis identified five low-dimension representations (LDRs) with correlation coefficients ranging between 0.14 (LDR5) and 0.67 (LDR1) (Figure S5).

**Figure 2:**
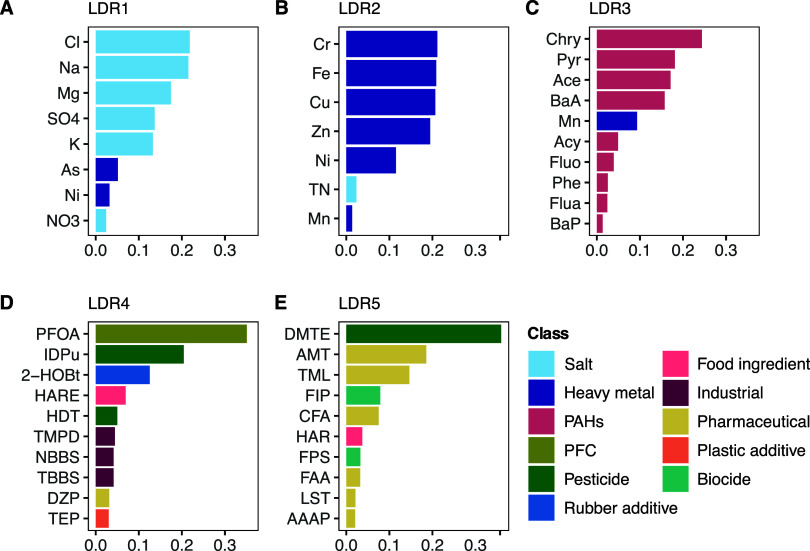
Chemical substance contributions to individual low-dimensional representation (LDR). These chemicals are ranked according to the squared weights (SW) and color-coded by their chemical classes. The names and chemical classes of these chemical substances are described in Table S2. Only chemical substances with squared weights greater than 0.01 are shown. The details of SWs are listed in Table S3.

Based on the relative contribution (squared weight, SW) of individual chemical features in each LDR, 44 chemical substances with SW more than 0.01 contributed to the SGCCA model, including nine inorganic chemicals in LDR1, six heavy metals in LDR2, nine PAHs in LDR3, ten polar organic chemicals in LDR4, and a different set of ten polar organic chemicals in LDR5 (Figure 2). The details of the SWs for these 44 chemical substances are listed in Table S3. Notably, these 44 chemical substances together explain 46.4% of the total variance in transcriptomic data, which was 86.7% of the variance explained by 215 chemical substances used in the SGCCA model. The chemical substances selected in LDR4 explained the highest amount (21.8%) of the total variance of the bioactivity profiles, followed by LDR 5 (14.3%), LDR3 (4.1%), LDR2 (3.1%) and LDR1 (3.0%) (Figure S5). These patterns suggest that these 44 chemical substances may be the driving factor of gene expression responses in exposed *D. magna* in this study, with organic chemical substances being the primary contributor. While the rest may subtly influence the daphnid transcriptome, it is premature to determine whether such chemicals pose discernible hazards to exposed daphnids and may future experimental investigation.

Based on the SW of individual genes, we performed a module enrichment analysis within LDRs. Twenty-one coexpression modules showed significant enrichment within one or more LDRs (adjusted *P*-value <0.05) (Figure S6). Four coexpression modules (modules 5, 7, 8, and 10) were shared across the LDRs. Eight modules were shared between LDR1 and LDR2, and eight modules were shared among LDR3, LDR4 and LDR5. Modules 11 and 21 were unique to LDR4, whereas module 12 was unique to LDR5 (Figure S6).

**Figure 3:**
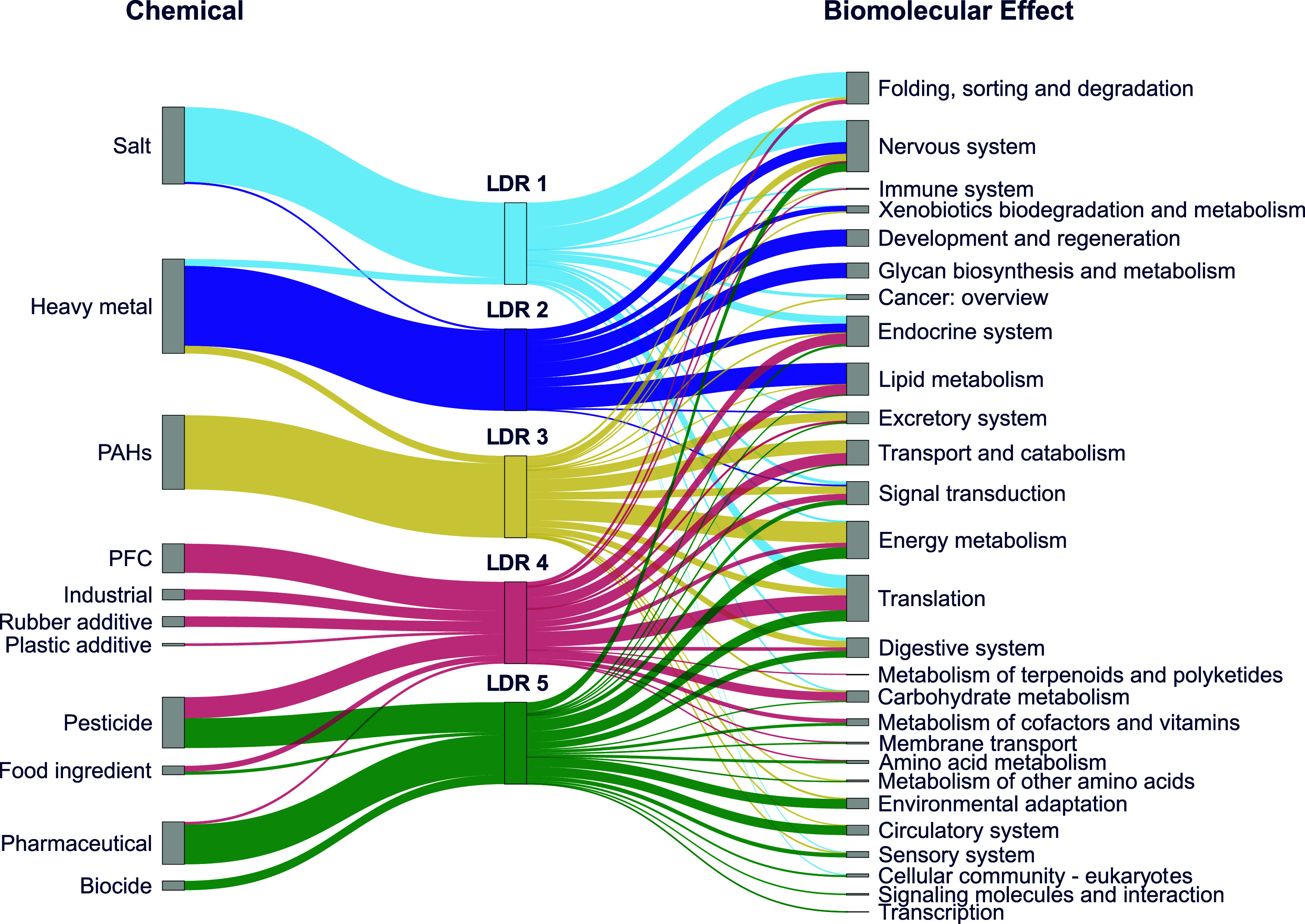
Biomolecular effects associated with individual low-dimensional representation (LDR). The correlation analysis (SGCCA) identifies five correlations between chemicals and bioactivity profiles. The five LDRs are coded by color. The strength of correlation is determined by chemical classes’ sums of squared weights (SWs) listed in Table S3.

We conducted a pathway overrepresentation analysis to identify significantly enriched pathways in the modules contributing significantly to each LDR. Five LDRs were associated with 80 KEGG pathways, which were categorized into 28 biomolecular effect categories (Figure 3 and Table S4). Five categories of biomolecular effects were shared among LDRs (LDR3-LDR5), including “carbohydrate metabolism”, ‘folding, sorting, and degradation’, “transport and catabolism”, “energy metabolism”, and “digestive system”. This pattern suggests that chemical mixtures may induce multiple biomolecular effects on distinct biological processes. Furthermore, top-ranked pathways are distinctive among these three LDRs (Table S5), which suggests that similar hazards can be associated with distinctive metabolic pathways. Besides, it is necessary to compare the bioactivity profiles of individual compounds with the bioactivity profiles of the chemical mixtures identified by this data-driven model to further determine whether synergistic interactions among chemicals within the mixture are occurring.^39,40^ Synergistic interactions may induce distinct pathways than those expected from exposures to individual chemicals.^41,42^ Furthermore, we cannot exclude that additional chemicals that were not quantified within our study from having contributed to the observed bioactivity profiles.

### Bioactivity Signatures of Three Organic Chemical Mixtures

We benchmarked and evaluated the pathway-level bioactivity signatures of three organic chemical mixtures from LDR3–5 using a comprehensive review of published studies. A total of 78 relevant papers were identified through a systematic search using keywords including the specific chemicals, “*Daphnia*,” “gene/transcript,” and “pathway.” These studies provided critical insights into the links between the chemicals and their biomolecular functions in *Daphnia*. When *Daphnia*-specific studies were unavailable, we supplemented our analysis with studies of other species to elucidate the chemical modes of action. This approach allowed us to infer potential mechanisms of toxicity and molecular interactions, providing a broader context for interpreting the observed pathway-level bioactivity detected in this study.

**Figure 4:**
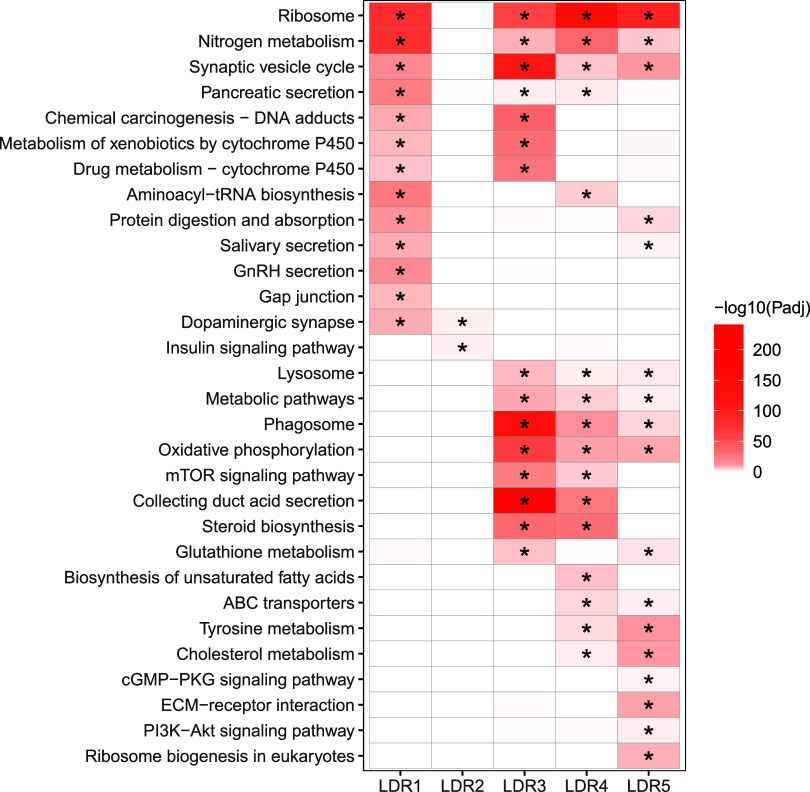
Pathway-level bioactivity signatures of individual low-dimensional representation (LDR). The pathway overrepresentation analysis evaluates the significance of enriching a specific pathway in significantly enriched modules (Figure S6) per LDR. These 30 pathways were selected based on their adjusted P-values. The log10 transformed adjusted P-values (Padj) of the chi-square testing significant correlations after FDR correction are shown. Asterisks (*) indicate pathways that are significantly enriched in an LDR based on Padj <0.05.

In **LDR3**, we identified 26 enriched pathways (Table S5) associated with nine PAHs, i.e., Chry, Pyr, Ace, BaA, Acy, Fluo, Phe, Flua, and BaP. The top-ranked pathways in this LDR comprise the synaptic vesicle cycle, oxidative phosphorylation, chemical carcinogenesis (forming DNA adducts), steroid biosynthesis, metabolism of xenobiotics by cytochrome P450 (CYP), drug metabolism, lysosome, and glutathione metabolism (Figure 4). PAH in LDR3 correlated with xenobiotic metabolism, oxidative stress, carcinogenesis, lipid metabolism and nervous system functions. CYPs are the functional enzymes in phase I of xenobiotic metabolism. Among these, there are the CYP monooxygenases involved in the biotransformation of PAHs in *Daphnia*.^43^

Enzymes observed in LDR3 and linked to PAHs have been previously associated with redox responses and phase II xenobiotic metabolism,^44^ including *D. magna*.^45^ The pathway related to the formation of DNA adducts, which we identified in the top-ranked pathways, has been previously linked to carcinogenesis.^46^ The perturbation in lipid metabolism observed in our study has been previously associated with chronic PAH toxicity.^47^

Previous studies have shown that BaP exposure may induce neurotoxicity in aquatic invertebrates^48,49^ by inhibiting acetylcholinesterase (AChE) and choline acetyltransferase (ChAT) activities.^50^ Although the cholinergic synapse pathway, which includes AChE and ChAT, was not significantly enriched in LDR3; AChE, a key biomarker in these enriched pathways, was a member of enriched modules. This finding suggests that the PAH mixture induces neurotoxicity by affecting AChE activity, which is crucial for terminating neurotransmission through the breakdown of acetylcholine released during the synaptic vesicle cycle.

In this LDR, we also identified novel pathways of toxicity. For example, a pathway regulating cardiac muscle contraction and three pathways critical for maintaining normal cardiac muscle contraction (aspartate β-hydroxylase, cytochrome c oxidase subunit 6b, and voltage-dependent calcium channel α-2/delta-1) were among the top-ranked pathways. Perturbation of these pathways could lead to severe cardiac dysfunctions and suggests the hazards posed by PAH mixtures can be more severe than those from single PAH exposures.^51^ This finding agrees with a previous study showing the enhanced effects of PAH mixtures in *D. magna*.^52^

In **LDR4**, we identified 35 enriched pathways (Table S5) correlated with nine organic polar compounds, including PFOA, pesticides (imidacloprid-urea, 2-hydroxyatrazine), a rubber additive (2-hydroxybenzothiazole), a food ingredient (harmine), three industrial chemicals (2,2,6,6-tetramethyl-4 piperidone, *n*-butylbenzenesulfonamide, 4-*tert*-butylbenzenesulfonamide), a pharmaceutical (diazepam), and a plastic additive (triethylphosphate). These pathways include steroid biosynthesis, biosynthesis of unsaturated fatty acids, fatty acid metabolism, mTOR signaling pathway, ABC transporters and PPAR signaling pathways, which have been previously associated with PFOA in rodents.^53−55^ Specifically, the MoAs of PFOA have been linked to the deregulating PPARα-dependent signaling pathway,^53^ the increased expression of mTOR that controls lipid synthesis,^54^ and the inhibition of cholesterol biosynthesis via the downregulation of the 3-hydroxy-3-methylglutaryl-CoA reductase and acyl CoA cholesterol acyltransferase.^55^ Studies of *Daphnia*’s responses to PFOA are limited, and a comprehensive overview of the pathway-level bioactivity signature of PFOA was not yet possible.^56^ While we recognize that there are major differences between mammals and invertebrates,^57^ this bioactivity signature identified in our study indicates the putative biomolecular targets in *Daphnia*.

Furthermore, we also identified a correlation between imidacloprid-urea (IDPu), a degradation product of a neurotoxic insecticide (imidacloprid) that acts on nicotinic acetylcholine receptors, and the synaptic vesicle cycle pathway^58^ (Figure 4), suggesting that IDP impairs the *Daphnia* nervous system.^59^ This chloronicotinyl insecticide is consistently detected in all downstream sites (Chaobai River, Table S2) and is commonly detected in aquatic environments.^59^ Additionally, IDPu has been found to suppress terpenoid backbone biosynthesis and fatty acid metabolism,^60^ which were also observed in our study.

In **LDR5**, we identified 38 enriched pathways (Table S5), including tyrosine metabolism, synaptic vesicle cycle, cholesterol metabolism, glutathione metabolism, PI3K-Akt signaling pathway, and cGMP-PKG signaling pathway (Figure 4). These pathways were associated with a pesticide metabolite (dimethachlor ESA), a biocide and its metabolite (fipronil and fipronil sulfide), a food ingredient (harman), and six pharmaceuticals (amantadine, tramadol, clofibric acid, losartan, n-acetyl-4-aminoantipyrine, and n-formyl-4-aminoantipyrine) (Figure 4). Within this bioactive mixture, dimethachlor ESA (a metabolite of the chloroacetanilide herbicide dimethachlor), fipronil (phenylpyrazole insecticide), and fipronil sulfide (a transformation product of fipronil) are GABA antagonists and obstruct the ion channel. Exposure to these pesticides can reduce GABA release, disrupting intraneural signal transduction and thereby suppressing neurotransmitter release.^61^ Our study also identified four pathways that were previously linked to cardiac and muscular dysfunctions. Organochlorine pesticides^62^ and fipronil^63^ were previously linked to irregular heart rates in *Daphnia*.

In addition, clofibric acid, as a metabolite of clofibrate, targets cholesterol metabolism to promote fatty acid metabolism. This agrees with the pathways identified in our study, such as cholesterol metabolism and fat digestion and absorption.^64^ Furthermore, the renin-angiotensin system pathway related to the endocrine system was identified in LDR5. This pathway is involved in the modulation of angiotensin peptides. Dysregulation of these enzymes can lead to angiotensin II and III imbalances, potentially causing inflammation or even organ damage.^65^ As losartan is an angiotensin II receptor blocker, it specifically inhibits the action of angiotensin II by blocking its binding to the AT1 receptor,^66^ which could be involved in the modulation of the renin-angiotensin system.

### Advantages and Limitations of the Data-Driven Approach

A key advantage of the data-driven approach used in this study is its ability to identify hazards induced by real-world chemical mixtures without bias. Unlike traditional methods that are hypothesis-driven and test chemicals at concentrations that the organisms rarely encounter in the natural environment, our approach examines biomolecular signatures induced by sublethal concentrations of ambient chemical mixtures. These bioactivity signatures can serve as early warning indicators of potential hazards, allowing protective measures to be taken. The multiblock correlation analysis that we employed had reduced the chemical complexity of environmental water samples to five components that have distinct pathway-level bioactivity signatures. Some signatures are indicative of known toxicity pathway and known adversities, yet here are associated with the potential MoAs of a combination of chemicals. Although it is not possible (not necessary) to discern how the chemicals interact as a component of the total mixture to produce their distinct biological effects, this approach significantly reduces the need for extensive testing across hundreds of chemicals. This is particularly advantageous since most chemicals from domestic and industrial sources are released into the environment as unintended mixtures with largely unknown toxicities.^67^ By linking chemicals to specific biomolecular signatures, our approach supports high-throughput screening of potential environmental hazards, identifying likely toxicity targets for further experimental validation.^14^ Specifically, in each LDR, we could associate biomolecular functions with potential chemical hazards within the ambient mixtures. Therefore, this methodology serves as an initial, unbiased screening tool for unintentional (thus unknown) hazards, potentially improving the efficiency and throughput of environmental risk assessments when routinely applied.

Our approach has its limitations. We selected the 48 h time point for this study because it aligns with the time frame used to measure ecological end points commonly adopted by regulatory agencies for risk assessment.^24^ This provides a practical and relevant foundation for benchmarking our data-driven approach against established ecological metrics. By targeting this regulatory benchmark, we aimed to ensure the translational relevance of our findings, particularly in the context of environmental decision-making frameworks. Conversely, early onset molecular events, such as gene expression changes, can occur before measurable ecological impacts become evident. These early responses can serve as sensitive indicators of chemical exposure and potential stressor impacts, offering the advantage of early warning signals in environmental monitoring.^14^ Integrating such early biomolecular data with apical end points could improve predictive modeling of long-term or population-level effects, providing a more proactive approach to risk assessment. Future work could benefit from incorporating multiple time points to capture the temporal dynamics of molecular responses^68^ and their correlation with ecological end points. Such an approach would allow us to refine our data-driven framework, potentially enhancing its sensitivity and predictive capacity while broadening its applicability across different regulatory contexts.

Another limitation of our study is that identifying correlations between biomolecular signatures and chemical mixtures does not necessarily establish causality. The associations discovered by our approach require future experimental validation using surrogate animal species to move from correlation to causation.^69^ The validation of these observations are crucial for confirming the biological relevance and potential environmental impact of the identified correlations.

In addition to these limitations, adopting the novel methodologies proposed in this study will require adjustments in regulatory frameworks, including phases of testing, validation, and acceptance by regulatory bodies. Despite these challenges, our data-driven approach has the potential to be transformative by reducing the need to conduct “forward” testing regimes, where first bionart, then trinary and higher-order mixtures are tested to assess the chemical mixture effects. Otherwise, these combinatorial tests are intractable at the scale of real-world complex effluents composed of hundreds or thousands of distinct substances. More importantly, it enables the generation of bioactivity signatures that enhance the mechanistic understanding of the hazardous effects of environmental chemical mixtures and their MoAs.

## Data Availability

Chemical fingerprinting data (target and target screening analysis) are listed in the Supporting Information Table S2. Raw transcriptomics data are submitted to NCBI (PRJNA809147).

## Supporting Information Available

Section A (Detailed methodology); Section B (Six supplementary figures: PCA plots for chemical and bioactivity profiles; Chemical fingerprinting; Diagnostic plots of gene coexpression network analysis; Visualizations of multiblock correlation analysis; Module enrichment analysis); Section C (Five supplementary tables: Description of sampling sites; Description of chemical fingerprinting; Square weight of individual chemicals in multiblock correlation analysis; Biomolecular effect profiles; Pathway profiles) (PDF)
Table S1. Overview of the sampling sites along the Chaobai River Basin; Table S2. Description of the analytical chemical fingerprints; Table S3. The relative contribution of individual chemical substance to each LDR; Table S4. Biomolecular effect of each LDR; Table S5. Bioactivity signature of each LDR (XLSX)
