# Supplementary material for: Bioactivity Profiling of Chemical Mixtures for Hazard Characterization
Source: Environ Sci Technol. 2024 Dec 20;59(1):291–301. doi: 10.1021/acs.est.4c11095 (PMC11741114; doi:10.1021/acs.est.4c11095)
Supplement: Supplementary file 1 — es4c11095_si_001.pdf [file es4c11095_si_001.pdf]

Supplementary Information

Summary: 10 pages, three sections of methods, six figures, five tables (in an Excel file)

Bioactivity Profiling of Chemical Mixtures for Hazard Characterisation

Xiaojing LI[1*], Jiarui Zhou[1*], Yaohui Bai[2], Meng Qiao[2], Wei Xiong[2], Tobias Schulze[3], Martin Krauss[3], Timothy D. Williams[1], Ben Brown[4], Luisa Orsini[1, 5§], Liang-Hong Guo[6§] and John K. Colbourne[1§]

1 Centre for Environmental Research and Justice (CERJ), School of Biosciences, the University of Birmingham, Birmingham, B15 2TT, U. K.

2 Research Centre for Eco-Environmental Sciences, Chinese Academy of Sciences, Beijing, 100085, P. R. China

3 Key Laboratory of Environmental Biotechnology, Research Centre for Eco-Environmental Sciences, Chinese Academy of Sciences, Beijing, 100085, P. R. China

4 Department Exposure Science, Helmholtz Centre for Environmental Research – UFZ, 04318 Leipzig, Germany

5 Environmental Genomics and Systems Biology Division, Lawrence Berkeley National Laboratory, Berkeley, 94720, U. S. A.

6 The Alan Turing Institute, British Library, London, NW1 2DB, U. K.

7 Hangzhou Institute for Advanced Study, UCAS, Hangzhou, Zhejiang 310020, P. R. China

Supplementary information includes three sections:

Section A. Supplementary Methods

Section B. Supplementary Figures

Section C. Supplementary Tables

Section A. Supplementary Methods

Targeted analysis of PAHs in the Chaobai River samples

Water sample (four litre) was firstly filtered through 0.7 μm glass fibre membrane filters (GF/F Whatman, U.S.A.). Internal standards (100 ng/L; 2-fluorobiphenyl and decachlorobiphenyl) were added before solid phase extraction (SPE). Cartridges used for SPE were C18 cartridges (500mg, 6ml, Supelco) and HLB cartridges (500mg, 6ml, Waters), then eluted with 10 ml dichloromethane and 5 ml hexane, successively. Sixteen PAHs were analysed using an Agilent 7890A gas chromatography (GC) with a 5795C mass spectrometry (MS) detector with electrospray ionisation (EI) in selective ion monitoring mode, as described by (Qiao et al., 2017, 2020).

Target screening of polar organic chemicals in the Chaobai River samples

The target screening analysis quantified polar organic chemicals, including pharmaceuticals, pesticides, biocides, and industrial chemicals. For this analysis, two litres of surface water were filtered over a 0.7 μm glass microfiber membrane (GF/F, Whatman) and extracted with HLB cartridges (500 mg, 6 mL, Waters) pre-conditioned with methanol and deionised water. A two-step elution was performed on these cartridges: (1) eluting with 5 ml 100 % methanol and 1 % formic acid, and then eluting with 5 ml 100 % methanol and 2 % 7N ammonia; (2) subsequently, eluting with 4 ml formic acid and subsequently 4 ml 7N ammonia in methanol. Elution both steps were then pooled in a single 50 ml glass vial and dried under mild nitrogen blow at room temperature.

The dried extracts were reconstituted at a relative enrichment factor of 500 (i.e. 500 mL of water correspond to 1 mL of extract) in methanol: H_2O, 70:30. Prior to analysis, a mixture of 40 isotope-labelled internal standards at a nominal concentration of 50 ng/mL in vial was spiked. Additionally, two sample preparation blanks were prepared from 130 μL methanol, 60 μL H2O and 10 μL of the internal standard mixture. All extracts were analysed using liquid chromatography-high resolution mass spectrometry (LC-HRMS) using a Thermo Ultimate 3000 LC system coupled to a quadrupole-Orbitrap instrument (Thermo QExactive Plus) with electrospray ionisation. LC separation was done on a Kinetex C18 EVO column (50 × 2.1 mm, 2.6 μm particle size, Phenomenex, pre-column 4 × 2.1 mm and in-line filter 0.2 μm) using a gradient elution with 0.1% of formic acid (eluent A) and methanol containing 0.1 % of formic acid (eluent B) at a flow rate of 300 μL/min. After 1 min of 5% eluent B, the fraction of eluent B was linearly increased to 100 % within 12 min and 100 % eluent B were kept for 11 min.

The eluent flow was diverted to waste and the column was rinsed for 2 min using a mixture of isopropanol and acetone (50:50) (mixture / eluent B / eluent A, 85 % / 10 % / 5 %) to remove hydrophobic matrix constituents from the column. Finally, the column was re-equilibrated to initial conditions for 5.7 min. The injection volume was 5 μL and the column was operated at 40 °C. Two separate runs were conducted in positive and negative ion mode, each combining a full scan experiment (100-1500 m/z) at a nominal resolving power of 70,000 (referenced to m/z 200) and data-independent MS/MS experiments at a nominal

resolving power of 35,000 with 12 different isolation windows. Those 12 broad isolation windows were 50 mu (i.e., m/z ranges 97-147, 144-194, 191-241, 238-288, 285-335, 332-382, 379-429, 426-476) and 260 mu (i.e., m/z ranges 473-733, 729-989, 985-1245, 1241-1501).

Data conversion of raw mass spectral mzML and centroiding was performed using ProteoWizard with the build-in instrument's library (Chambers et al., 2012). Peak picking, alignment, gap filling and peak annotation was done in MZmine 2.52 (http://mzmine.github.io). The resulting annotated peak list was further analysed using an in-house R-package MZquant (version 0.7.22) for semi-automated quantification. The steps included final clean-up of the annotations, blank filtering, automated assignment of internal standards and quantification of the targeted peaks. Compounds with very broad peaks or high background noise, which could not be quantified by this semi-automatic workflow, were analysed manually with the vendor software Tracefinder 4.1 (Thermo Scientific). The raw peak tables were refined by the final peak annotations after Mzquant and Tracefinder assessment.

Diagnostic plots of co-expression network

Figure S3 (session B below) depicts the process of determining the cutoff value of Pearson pairwise correlation coefficients (coef). This will ensure that the topology of the resulting co-expression network follows a scale-free model. By testing various hard thresholding of coef, when the threshold set at 0.60, the resulting network has the scale free topology.

Reference

Chambers, M. C., Maclean, B., Burke, R., Amodei, D., Ruderman, D. L., Neumann, S., Gatto, L., Fischer, B., Pratt, B., Egertson, J., Hoff, K., Kessner, D., Tasman, N., Shulman, N., Frewen, B., Baker, T. A., Brusniak, M.-Y., Paulse, C., Creasy, D., … Mallick, P. (2012). A cross-platform toolkit for mass spectrometry and proteomics. *Nature Biotechnology*, *30*(10), 918–920. https://doi.org/10.1038/nbt.2377

Qiao, M., Cao, W., Liu, B., Zhao, X., & Qu, J. (2017). Simultaneous detection of chlorinated polycyclic aromatic hydrocarbons with polycyclic aromatic hydrocarbons by gas chromatography–mass spectrometry. *Analytical and Bioanalytical Chemistry*, *409*(13), 3465–3473. https://doi.org/10.1007/s00216-017-0290-1

Qiao, M., Fu, L., Li, Z., Liu, D., Bai, Y., & Zhao, X. (2020). Distribution and ecological risk of substituted and parent polycyclic aromatic hydrocarbons in surface waters of the Bai, Chao, and Chaobai rivers in northern China. *Environmental Pollution*, *257*, 113600. https://doi.org/10.1016/j.envpol.2019.113600

Section B. Supplementary Figures

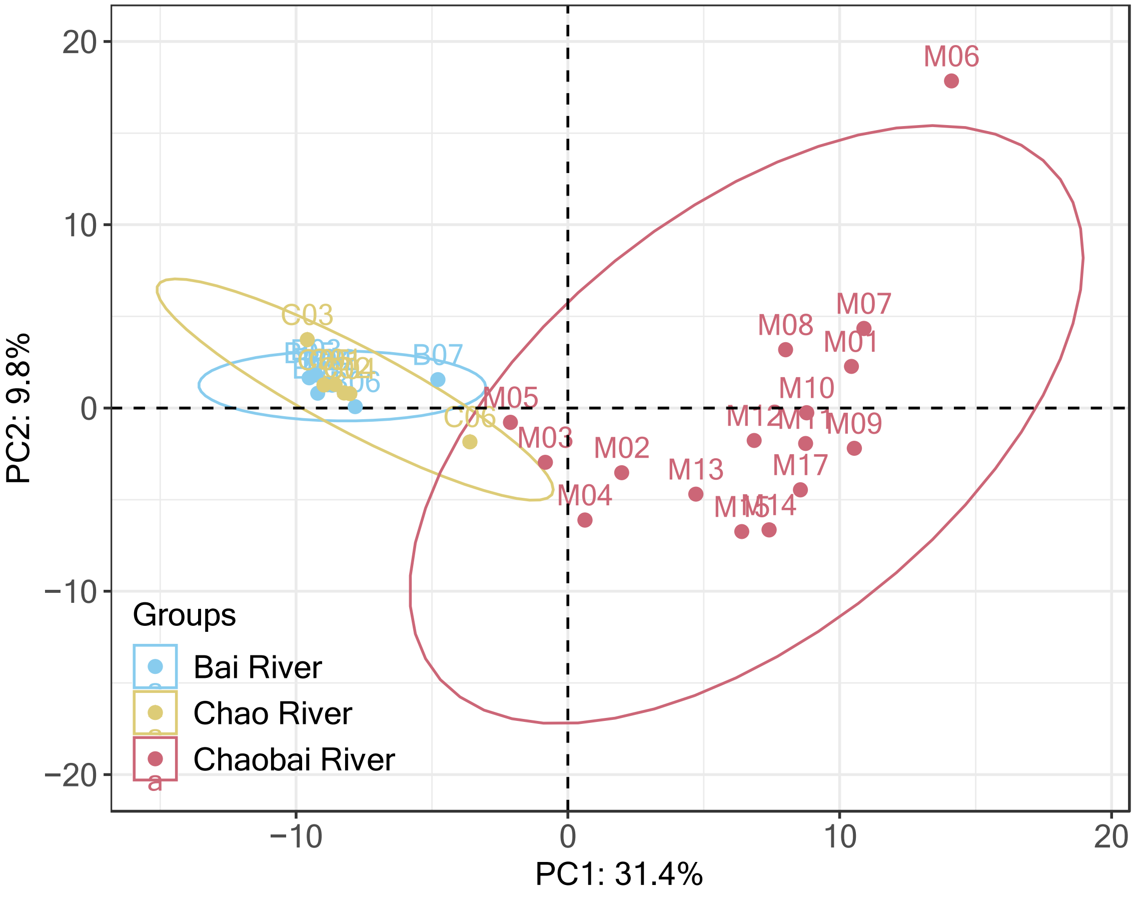

Figure S1. This principal component analysis (PCA) plot is generated based on the chemical fingerprinting data, which displays individual sampling sites as dots, colour-coded by river reach: Bai River (blue), Chao River (yellow), and Chaobai River (red). The proportion of variance explained by the first two components is indicated on the x- and y-axis labels.

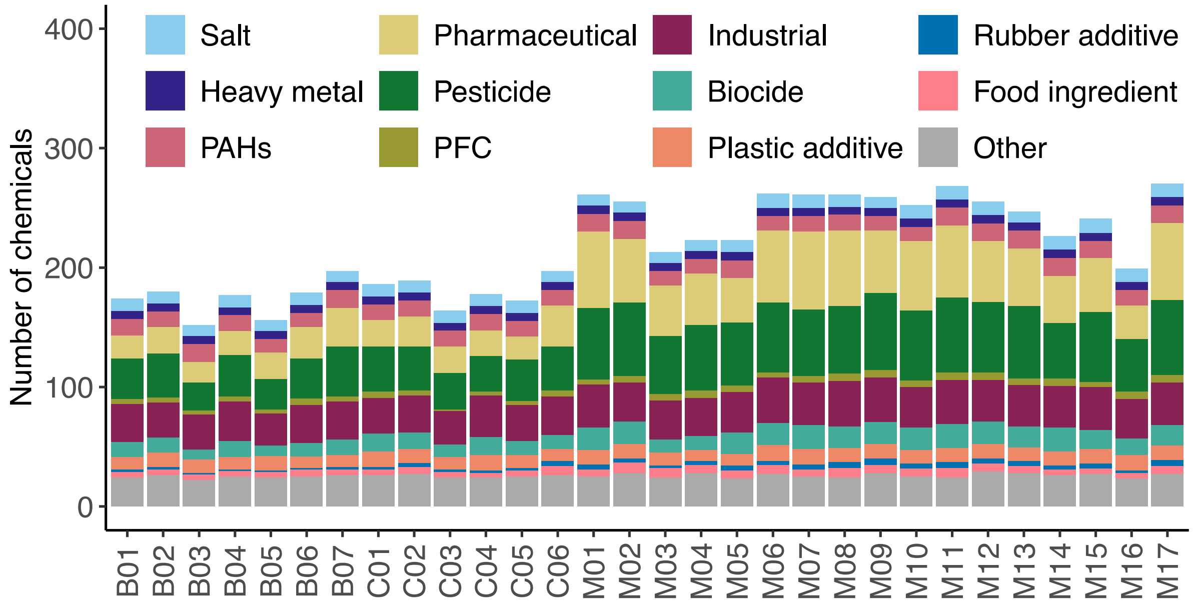

Figure S2. Chemical fingerprinting of water samples from the Chaobai River. The site names on the x-axis indicate different river reaches, such as B for Bai River, C for Chao River, and M for Chaobai River; the numbers are the sequentially positioned sampling sites along the river, from upstream to downstream (Table S1). The bar plot shows the total number of chemicals identified per site, colour-coded by the chemical usage groups described in Table S2. The chemical class "Other" includes surfactants, UV filters, flame retardants, stimulants, fragrances, sweeteners, repellents, corrosion inhibitors, human metabolites, natural compounds, bittern and dyes.

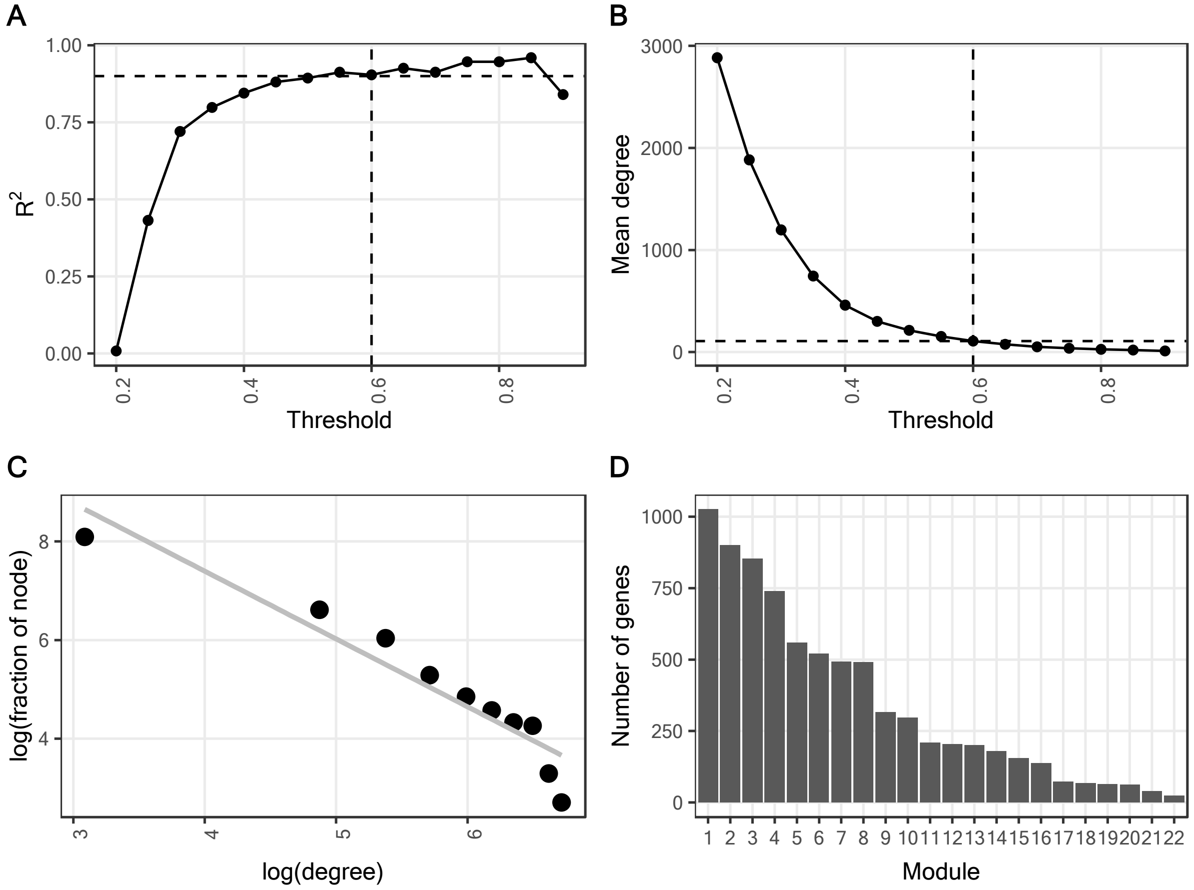

Figure S3. Gene co-expression network. The expressions of all the genes were used to compute the pairwise Pearson correlation coefficient (coef). (A) Analysis of the scale-free fit index for various hard thresholding of coef. (B) Analysis of the mean degree for various hard thresholding of coef. (C) Checking the scale free topology when cutoff = 0.60. (D) A total of 22 co-expression modules were identified in this study. The bar plot indicates the number of genes of each co-expression module.

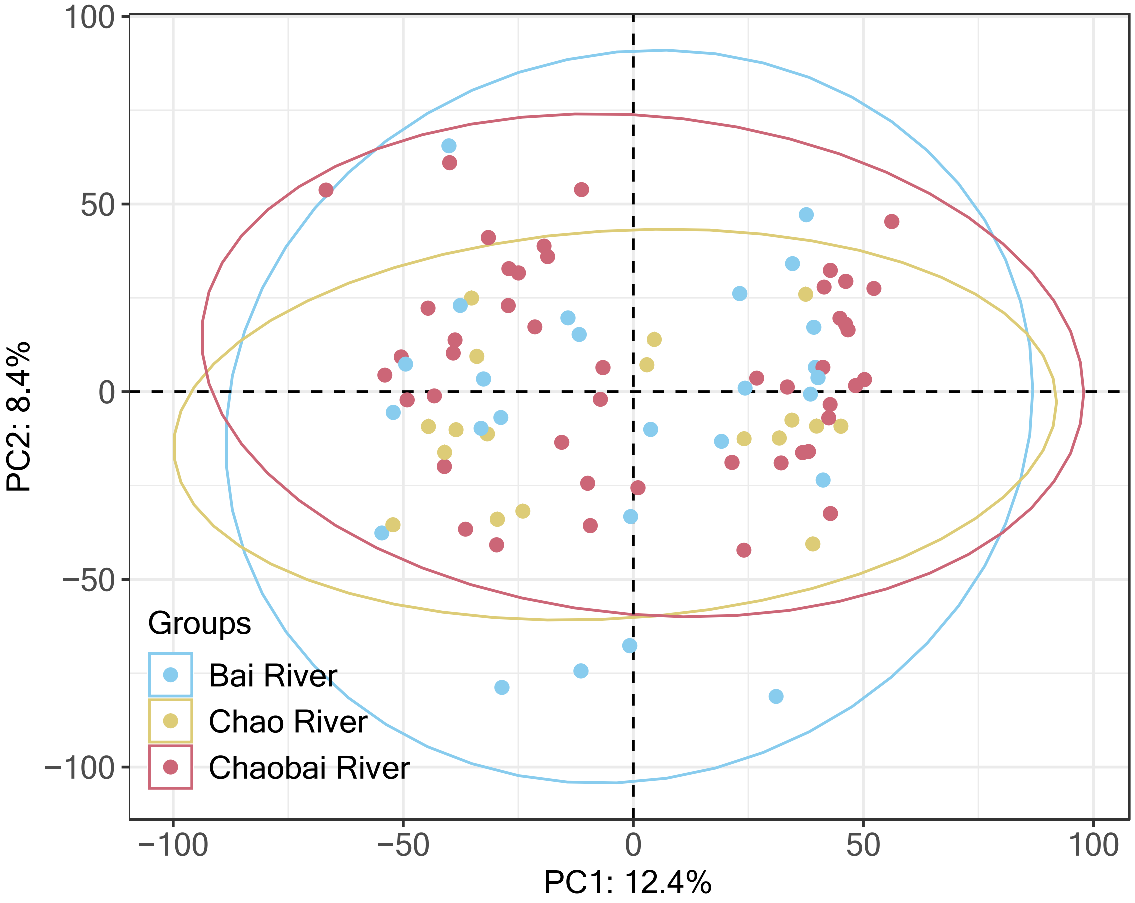

Figure S4. Overview of transcriptomic profiles of exposed daphnids in the Chaobai case study. PCA plot illustrates the general similarity among transcriptomic profiles of exposed daphnids in this study. The individual dots represent different sampling sites, coloured by river reaches. The proportion of variance explained by the first two components is indicated on the x- and y-axis labels.

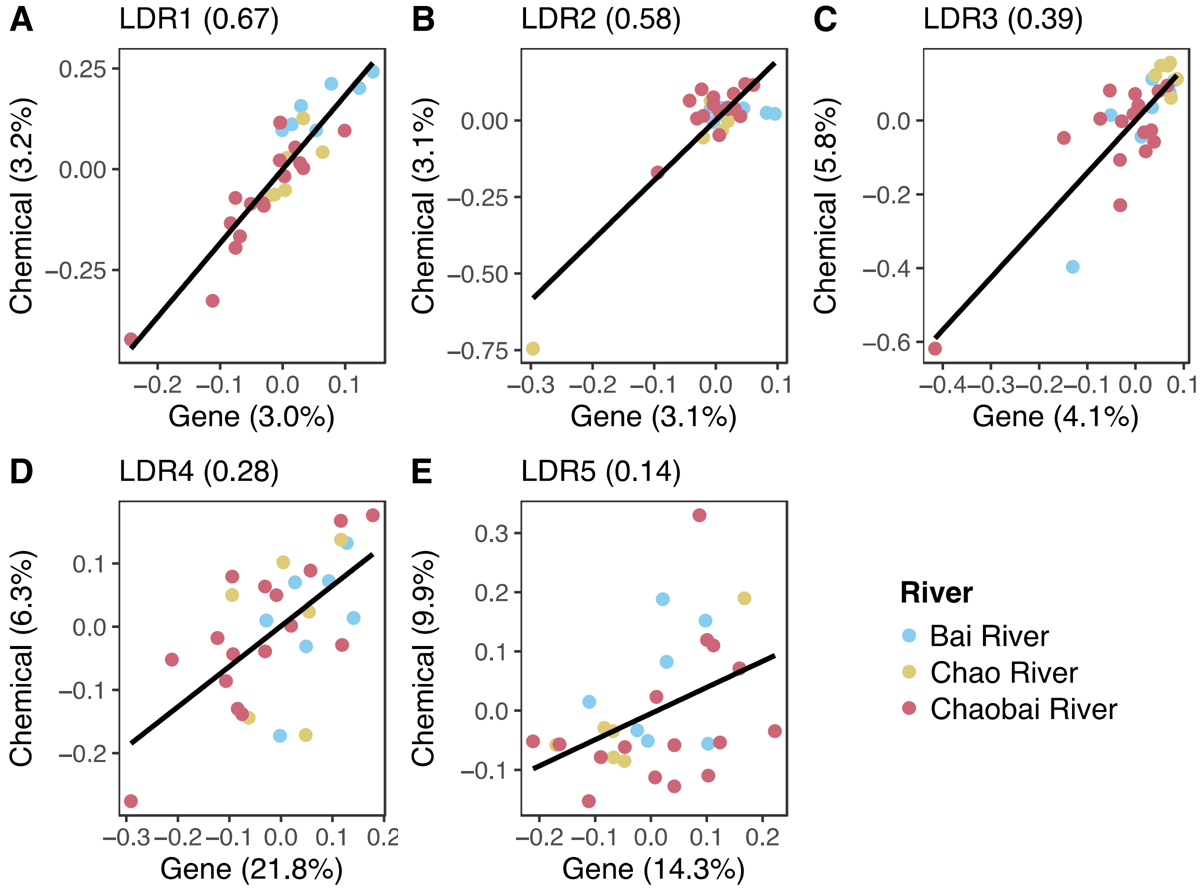

Figure S5. Multi-block correlation analysis by sparse generalized canonical correlation analysis (SGCCA). The concentrations of 215 chemical substances from the chemical fingerprinting and the expressions of 10440 genes detected in the bioactivity profiles of exposed Daphnia were used for multi-block correlation to reveal their relationships. Five paired low dimensional representations (LDRs) were obtained from the SGCCA model, with the variance explained by each LDR displaying on corresponding x and y axes. Single dot represent a sampling site, with colour-coded by river reach: Bai River, blue; Chao River, yellow; Chaobai River, red. The scatter plots for the five LDR pairs are (A) LDR1, (B) LDR2, (C) LDR3, (D) LDR4, and (E) LDR5.

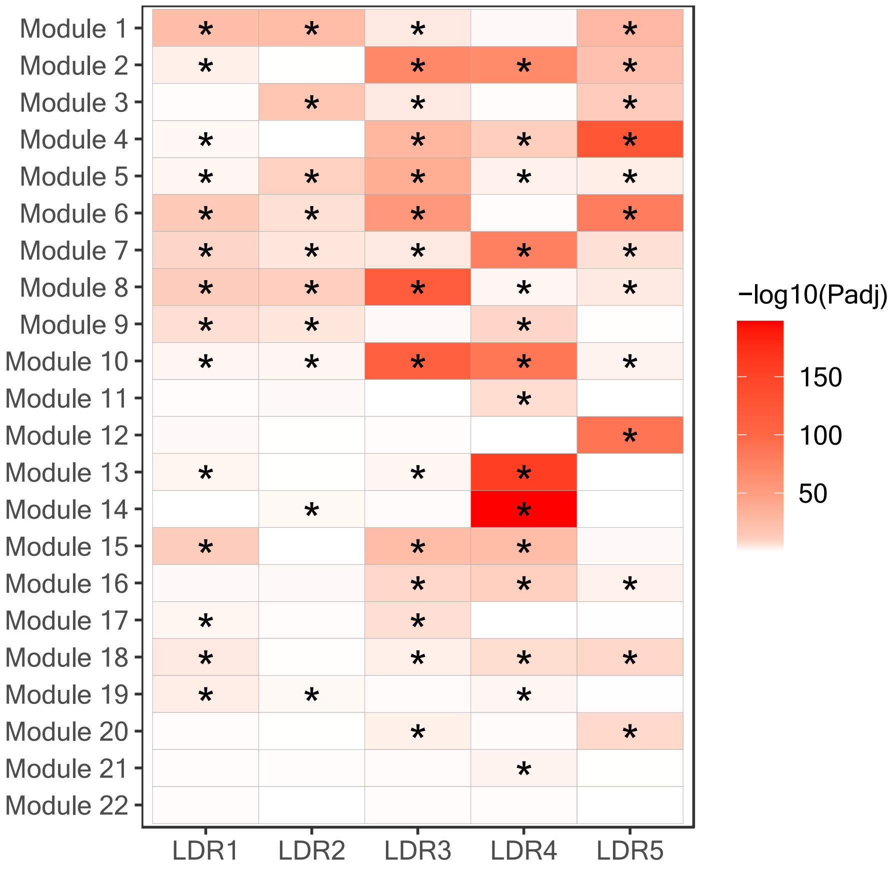

Figure S6. Module enrichment analysis. The colour coding is referred to minus log10 transformed adjusted P-value of Mann-Whitney U test after FDR correction. The asterisk (*) highlights the modules that are significantly enriched based on Padj < 0.05.

Section C. Supplementary Tables (in a separate Excel file)

Table S1. Overview of the sampling sites along the Chaobai River Basin. The GPS locations and on-site measurements (the pH and total dissolved solids) are summarised in this table.

Table S2. Description of the analytical chemical fingerprints. This table summarises the chemical names, CAS, chemical classes, units, number of detectable sites (occurrence of all 30 sites and among Bai, Chao, and Chaobai Rivers), minimum, average, and maximum concentrations, and the concentrations detected in individual sites.

Table S3. The relative contribution of individual chemical substance to each LDR. The squared weight (SW) of each chemical in the SGCCA model evaluates the relative contribution.

Table S4. Biomolecular effect of each LDR. The pathways listed in Table S4 are summarised by their biomolecular effect categories based on their occurrence in individual LDRs.

Table S5. Bioactivity signature of each LDR. With pathway overrepresentation analysis, the significantly enriched pathways of those significantly enriched co-expression modules (listed in Figure S5) were listed in this table, with adjusted P-value less than 0.05 highlighted in red (corrected by False discovery rate).
